# Correlates of leisure-time sedentary behavior among 181,793 adolescents aged 12-15 years from 66 low- and middle-income countries

**DOI:** 10.1371/journal.pone.0224339

**Published:** 2019-11-14

**Authors:** Davy Vancampfort, Tine Van Damme, Joseph Firth, Mats Hallgren, Lee Smith, Brendon Stubbs, Simon Rosenbaum, Ai Koyanagi

**Affiliations:** 1 KU Leuven Department of Rehabilitation Sciences, Leuven, Belgium; 2 KU Leuven, University Psychiatric Center KU Leuven, Kortenberg, Belgium; 3 NICM Health Research Institute, Western Sydney University, Sydney, Australia; 4 Division of Psychology and Mental Health, Faculty of Biology, Medicine and Health, University of Manchester, Manchester, United Kingdom; 5 Centre for Youth Mental Health, University of Melbourne, Melbourne, Australia Department of Public Health Sciences, Melbourne, Australia; 6 Karolinska Institutet, Stockholm, Sweden; 7 Cambridge Centre for Sport and Exercise Sciences, Anglia Ruskin University, Cambridge, United Kingdom; 8 Physiotherapy Department, South London and Maudsley NHS Foundation Trust, Denmark Hill, London, United Kingdom; 9 Department of Psychological Medicine, Institute of Psychiatry, Psychology and Neuroscience, King's College London, De Crespigny Park, London, United Kingdom; 10 School of Psychiatry, University of New South Wales, Sydney, Australia; 11 Black Dog Institute, Prince of Wales Hospital, Sydney, Australia; 12 Research and Development Unit, Parc Sanitari Sant Joan de Déu, Universitat de Barcelona, Fundació Sant Joan de Déu, CIBERSAM, Barcelona, Spain; 13 ICREA, Pg. Lluis Companys 23, Barcelona, Spain; University of the Witwatersrand, SOUTH AFRICA

## Abstract

**Background:**

Sedentary behavior is a growing public health concern in young adolescents from low- and middle-income countries (LMICs). However, a paucity of multinational studies, particularly in LMICs, have investigated correlates of leisure-time sedentary behavior (LTSB) in young adolescents. In the current study, we assessed socio-demographic, socio-economic, socio-cultural and health behavior related correlates of LTSB among adolescents aged 12–15 years who participated in the Global school-based Student Health Survey (GSHS).

**Methods:**

Self-reported LTSB, which was a composite variable assessing time spent sitting and watching television, playing computer games, talking with friends during a typical day excluding the hours spent sitting at school and doing homework, was analyzed in 181,793 adolescents from 66 LMICs [mean (SD) age 13.8 (1.0) years; 49% girls). Multivariable logistic regression was used to assess the potential LTSB correlates.

**Results:**

The overall prevalence of ≥3 hours/day of LTSB was 26.4% (95%CI = 25.6%-27.2%). Increasing age (OR = 1.14; 95%CI = 1.11–1.17), past 30-day smoking (OR = 1.85; 95%CI = 1.69–2.03), alcohol consumption (OR = 2.01; 95%CI = 1.85–2.18), and bullying victimization (OR = 1.39; 95%CI = 1.31–1.48) were positively associated with increased LTSB across the entire sample of 181,793 adolescents. Food insecurity (OR = 0.93; 95%CI = 0.89–0.97) and low parental support/monitoring (OR = 0.91; 95%CI = 0.85–0.98) were negatively associated with LTSB. There were some variations in the correlates between countries.

**Conclusions:**

Our data indicate that in adolescents aged 12 to 15 years living in LMICs, LTSB is a complex and multi-dimensional behavior determined by socio-demograhic, sociocultural, socio-economic, and health behavior related factors. Future longitudinal data are required to confirm/refute these findings, and to inform interventions which aim to reduce sedentary levels in adolescents living in LMICs.

## Introduction

There is growing interest in the harmful effects of sedentary behavior (i.e. any waking behavior characterized by an energy expenditure ≤1.5 METs while in a sitting, reclining or lying posture) [1] in adolescents. In adolescents, sedentary behavior is associated with a higher risk for physical and mental health conditions, such as cardio-metabolic disorders and depression [2–6]. These chronic non-communicable conditions are particularly burdensome in low- and middle-income countries (LMICs). For example, the prevalence of depression among adolescents in LMICs is as high as 28%[7], while almost 75% of non-communicable disease related deaths occur in LMICs [8].

This high risk indicates that there is a large potential for preventive interventions such as reducing time spent sedentary at the early stages of life in this part of the world [8], in particular, based on the fact that behavioral patterns formed during adolescence can carry over into adulthood [9]. There is a large body of evidence, which suggests that decreasing any type of sedentary time is associated with lower health risk in children and adolescents aged 5–17 years. In particular, the evidence suggests that daily TV viewing in excess of 2 hours is associated with greater physical health risks, and that lowering sedentary time leads to reductions in body mass index (BMI) [10].

Understanding barriers and facilitators of sedentary behavior in adolescents living in LMICs is an important first step to translate the existing evidence in real world settings. The focus to date on correlates associated with sedentary behaviors has mostly been on the individual level such as psychological, behavioral and biological correlates [11]. However, it has become apparent that these are not stand-alone correlates and addressing them in isolation will not result in a significant change in time adolescents spent sedentary [11]. Interpersonal, environmental and policy factors may also need to be taken into account when exploring correlates of sedentary behavior [12]. The current rationale is that correlates associated with sedentary behavior can be conceptualized within models such as the socio-ecological model [12]. This approach emphasizes the fact that multiple-level factors influence the time spent sedentary, and that focusing on the interrelationships between individual, interpersonal, environmental and policy factors are important [11].

Exploring correlates of sedentary behaviour at different levels of the socio-ecological model specifically in adolescents in LMICs is important as there may be differences in sociocultural attitudes towards sedentary behavior (e.g., using motorized transport as a sign of wealth) and different environmental factors (e.g., safety and climate issues that cause adolescents to be more sedentary) in comparison with high-income countries [13]. Further, the association between an adolescent’s socio-economic status and sedentary behavior, may differ between high-income countries (HICs) and LMICs. For example, while a higher socio-economic status has been associated with lower sedentary levels in high-income countries, the opposite has been reported in LMICs [14]. Differences in access to TVs and computer games may partly explain this finding, as previous research, mainly in HICs, has consistently found that adolescents with more access to TVs and computers report more screen-based sedentary behavior [15].

To date, multinational studies exploring sedentary behavior correlates in adolescents aged 12–15 years in LMICs are scarce. Multinational studies allow exploration of sedentary behavior correlates irrespective of national policies and available facilities, and at the same time allow comparison between countries in order to investigate the role of these policies and available facilities in different countries. Previous multinational studies in adolescents from LMICs have shown that sedentary behavior is associated with obesity [16], depression [17], loneliness [18], and fast food and carbonated soft drink consumption [19]. However, there is limited information on other important correlates from a global perspective.

Thus, in the current study, we assessed at the personal [age, gender and food insecurity as a measure of proxy for socio-economic status, and health-behavior related correlates (e.g., smoking, alcohol use)] and at the interpersonal (parental support, number of friends, bullying) level of the socio-ecological model, correlates of leisure time sedentary behaviour (LTSB) among adolescents aged 12–15 years who participated in the Global school-based Student Health Survey (GSHS). These variables are available in the GSHS dataset and were selected based on past literature [14, 20–23]. Based on previous literature [14, 20–23], we hypothesized that older age, male sex, a higher socio-economic status, lack of parental support, a low number of friends, and being bullied are all associated with being more sedentary in adolescents aged 12–15 years from LMICs.

## Methods

### The survey

Data from the Global school-based Student Health Survey (GSHS) were analyzed. These data are publicly available at https://www.who.int/ncds/surveillance/gshs/datasets/en/. The GSHS was developed by the United States Centers for Disease Control and Prevention, the World Health Organization and other United Nations allies. The survey draws content from the CDC Youth Risk Behavior Survey (YRBS) for which test-retest reliability has been established[24]. The survey used a standardized two-stage probability sampling design for the selection process within each participating country. In the first stage, schools were selected with probability proportional to size sampling, while in the second stage a random selection of classrooms took place within each selected school. All students in the selected classrooms were eligible to participate in the survey regardless of age. Data collection via self-administration was performed during one regular class period. The multiple-choice questionnaires were translated into the local language in each country. Responses were completed on computer scannable sheets. Ethical approval was in each country obtained from both a national government administration (most often the Ministry of Health or Education) and an institutional review board or ethics committee. Student privacy was protected through anonymous and voluntary participation, and informed consent was obtained from the students, parents and/or school officials. Data were weighted for non-response and probability selection.

Sixty-six nationally representative datasets from LMICs included the variables used in the current analysis. If there were more than two datasets from the same country, we chose the most recent dataset. For the included countries, the survey was conducted between 2003 and 2016, and consisted of 11 low-income, 33 lower middle-income, and 22 upper middle-income countries based on the World Bank classification at the time of the survey. The characteristics of each country or survey are provided in Table 1. Response rates for each country ranged from 60% (Senegal) to 100% (Jordan).

**Table 1:** Sample characteristics and prevalence of ≥3 hours/day of leisure-time sedentary behavior ^a^.

Country-income	Country	Year	Response rate (%)	N	LTSB ≥3h/day (%)
Low	Afghanistan	2014	79	1,493	23.3
	Benin	2016	78	717	25.2
	Cambodia	2013	85	1,812	10.2
	Kenya	2003	84	2,971	37.7
	Mozambique	2015	80	668	41.0
	Myanmar	2007	95	2,227	9.7
	Nepal	2015	69	4,616	9.8
	Senegal	2005	60	2,666	25.4
	Tanzania	2014	87	2,615	20.1
	Uganda	2003	69	1,904	27.4
	Zambia	2004	70	1,365	32.6
Lower middle	Bangladesh	2014	91	2,753	14.9
	Belize	2011	88	1,600	36.3
	Bolivia	2012	88	2,804	24.3
	Djibouti	2007	83	962	32.3
	East Timor	2015	79	1,631	15.6
	Egypt	2011	85	2,364	27.5
	El Salvador	2013	88	1,615	35.2
	Ghana	2012	82	1,110	18.4
	Guatemala	2015	82	3,611	22.9
	Guyana	2010	76	1,973	35.7
	Honduras	2012	79	1,486	30.3
	India	2007	83	7,330	22.8
	Indonesia	2015	94	8,806	24.5
	Jordan	2007	100	1,648	38.2
	Kiribati	2011	85	1,340	14.4
	Laos	2015	70	1,644	19.2
	Macedonia	2007	93	1,550	49.9
	Maldives	2009	80	1,981	42.4
	Mauritania	2010	70	1,285	38.9
	Mongolia	2013	88	3,707	39.6
	Morocco	2010	92	2,405	25.7
	Pakistan	2009	76	4,998	8.2
	Philippines	2015	79	6,162	30.7
	Samoa	2011	79	2,200	38.1
	Solomon Islands	2011	85	925	26.4
	Sri Lanka	2008	89	2,504	33.2
	Sudan	2012	77	1,401	19.7
	Syria	2010	97	2,929	25.3
	Tonga	2010	80	1,946	29.2
	Tunisia	2008	83	2,549	23.9
	Vanuatu	2011	72	852	19.0
	Vietnam	2013	96	1,743	34.9
	Yemen	2014	75	1,553	19.4
Upper middle	Algeria	2011	98	3,484	26.8
	Antigua & Barbuda	2009	67	1,235	54.6
	Argentina	2012	71	21,528	49.9
	Botswana	2005	95	1,397	34.6
	Costa Rica	2009	72	2,265	44.2
	Fiji	2016	79	1,537	28.9
	Grenada	2008	78	1,299	41.1
	Iraq	2012	88	1,533	25.6
	Lebanon	2011	87	1,982	47.2
	Libya	2007	98	1,891	28.6
	Malaysia	2012	89	16,273	42.7
	Mauritius	2011	82	2,074	39.2
	Namibia	2013	89	1,936	37.2
	Oman	2005	97	2,426	34.2
	Peru	2010	85	2,359	28.6
	Seychelles	2007	82	1,154	51.4
	St. Vincent & the Grenadines	2007	84	1,188	39.1
	St. Lucia	2007	82	1,072	52.6
	Suriname	2009	89	1,046	40.3
	Thailand	2015	89	4,132	50.7
	Tuvalu	2013	90	679	15.3
	Uruguay	2006	71	2,882	49.6

Abbreviation: LTSB Leisure-time sedentary behavior.

^a^ Based on students aged 12–15 years.

### Leisure-time sedentary behavior (LTSB)

LTSB was assessed with the question “How much time do you spend during a typical or usual day sitting and watching television, playing computer games, talking with friends, or doing other sitting activities?” with answer options: <1, 1–2, 3–4, 5–6, 7–8, and ≥8 hours/day. This excluded time at school and when doing homework. In accordance with previous research showing that engaging in sedentary behavior for ≥3 hours/day is associated with significant health risks [25], the variable was dichotomized (≥3 hours/day or not),

### Correlates

A total of eight potential correlates of sedentary behavior were selected based on past literature [14, 20–23].

#### Socio-demographic variables

The sociodemographic variables included age, sex, and socioeconomic status. Food insecurity was used as a proxy for socioeconomic status as there were no variables on socioeconomic status in the GSHS. It was assessed by the question “During the past 30 days, how often did you go hungry because there was not enough food in your home?” Answer options were categorized as ‘never/rarely’ (coded 0) and ‘sometimes/most of the time/always’ (coded 1).

#### Socio-cultural variables

Parental support/monitoring: Low parental support/monitoring was defined as answering ‘rarely’ or ‘never’ to all of the following three questions: (a) ‘during the past 30 days, how often did your parents or guardians check to see if your homework was done?’; (b) ‘during the past 30 days, how often did your parents or guardians understand your problems and worries?’; and (c) ‘during the past 30 days, how often did your parents or guardians really know what you were doing with your free time?’[26].

Close friends: referred to the number of close friends a student has. This variable was dichotomized into at least one (coded 0) and none (coded 1). Bullying victimization was defined as being bullied on at least one day in the past 30 days.

#### Other health risk behaviors

Smoking: referred to the use of any form of tobacco on at least one day in the past 30 days.

Alcohol consumption: was defined as having had at least one drink containing alcohol in the past 30 days.

### Statistical analysis

Multivariable logistic regression analysis was conducted to assess the association between each correlate (exposure) and LTSB (outcome) based on data from each country. The analysis was adjusted for age, sex, and food insecurity. The association of age, sex, and food security with LTSB was assessed with a model that mutually adjusted for these three variables. Not all countries could be included in some analyses as data on some variables were not collected from certain countries (See Table 2 for availability of data for each country). The Higgins’s *I*^*2*^ statistic was calculated in order to assess the level of between-country heterogeneity. A value of <40% is often considered as negligible and 40–60% as moderate heterogeneity[27]. Pooled estimates were obtained by combining the estimates for each country into a random effect meta-analysis (overall and by country-income level). All variables were included in the regression analysis as categorical variables with the exception of age. Taylor linearization methods were used in all analyses to account for the sample weighting and complex study design. Results from the logistic regression analyses are presented as odds ratios (ORs) with 95% confidence intervals (CIs). The level of statistical significance was set at p<0.05. Statistical analyses were performed with Stata 14.1 (Stata Corp LP, College station, Texas). Data analysis was conducted in June 2019.

**Table 2:** Prevalence or mean of the correlates by country.

Country-income	Country	Age	Male	FI	Smoking	Alcohol	Low PS/M	No friend	Bullied
Low	Afghanistan	14.0 (0.9)	53.4	37.0	9.1	NA	12.8	13.7	43.8
	Benin	14.2 (0.9)	65.6	26.5	5.2	38.6	14.9	11.8	48.4
	Cambodia	14.1 (0.8)	48.4	30.8	4.0	5.2	NA	5.7	22.1
	Kenya	13.9 (1.0)	47.5	48.2	20.0	16.3	7.5	12.4	58.3
	Mozambique	14.1 (0.8)	49.6	35.2	4.8	9.4	10.2	10.3	45.7
	Myanmar	13.6 (1.0)	49.5	28.6	4.5	0.9	4.1	3.8	20.0
	Nepal	13.8 (1.0)	47.3	27.3	7.0	4.6	12.3	4.4	50.3
	Senegal	13.9 (1.0)	60.2	26.7	7.1	3.0	13.8	NA	NA
	Tanzania	13.6 (1.0)	46.8	14.4	6.7	4.2	17.6	8.7	26.9
	Uganda	14.3 (0.8)	47.4	38.3	6.9	12.7	13.2	10.6	45.6
	Zambia	13.9 (1.0)	50.3	72.3	NA	43.1	10.9	15.7	65.0
Lower middle	Bangladesh	14.0 (0.8)	63.4	54.4	9.0	1.4	9.1	8.8	23.7
	Belize	13.6 (1.1)	48.4	25.2	NA	25.2	10.6	7.8	30.7
	Bolivia	14.0 (0.9)	49.7	26.1	14.1	14.7	19.8	8.2	30.4
	Djibouti	14.3 (0.8)	59.5	38.0	10.2	NA	13.4	NA	40.6
	East Timor	14.1 (1.0)	46.3	32.9	22.8	12.3	26.5	4.9	31.3
	Egypt	13.5 (0.9)	49.2	19.6	6.2	NA	12.6	8.2	70.1
	El Salvador	14.0 (0.9)	50.6	15.0	NA	16.7	12.6	5.2	22.5
	Ghana	13.8 (1.0)	49.1	55.6	16.7	15.3	11.3	10.0	62.8
	Guatemala	13.9 (0.9)	50.9	13.9	NA	16.6	NA	6.5	23.0
	Guyana	14.1 (0.8)	48.6	33.4	15.4	39.3	11.5	10.3	38.4
	Honduras	13.6 (1.0)	46.1	13.7	13.3	14.8	14.8	6.8	32.3
	India	13.9 (0.9)	57.4	16.6	4.0	NA	10.0	10.2	NA
	Indonesia	13.5 (1.0)	49.2	42.2	11.5	3.7	8.0	3.1	21.0
	Jordan	14.3 (0.7)	47.3	29.1	22.5	NA	14.4	8.2	41.3
	Kiribati	14.0 (0.9)	45.5	43.2	31.3	29.8	24.6	2.6	36.8
	Laos	14.5 (0.8)	47.8	29.8	3.9	19.8	19.3	5.1	13.2
	Macedonia	13.9 (0.9)	51.6	4.9	10.1	34.5	6.5	2.4	9.8
	Maldives	14.4 (0.7)	47.9	24.1	12.1	5.0	12.6	9.6	37.0
	Mauritania	14.2 (0.9)	53.2	26.5	24.1	NA	19.9	7.6	47.5
	Mongolia	13.7 (1.0)	49.4	13.3	8.3	4.1	14.6	6.0	31.4
	Morocco	13.7 (1.0)	52.9	22.3	8.9	NA	20.1	8.8	18.5
	Pakistan	14.1 (0.8)	60.8	18.9	10.1	NA	9.3	8.1	41.1
	Philippines	13.9 (0.9)	48.1	37.7	13.8	17.5	22.8	4.2	51.5
	Samoa	14.0 (0.8)	47.4	73.7	45.3	34.5	9.7	15.9	74.1
	Solomon Islands	14.1 (0.9)	52.1	73.7	28.5	17.6	8.3	13.4	65.7
	Sri Lanka	13.7 (0.9)	49.8	19.9	NA	NA	5.9	5.5	37.6
	Sudan	14.2 (0.8)	51.9	22.0	10.2	NA	14.6	NA	NA
	Syria	13.6 (1.0)	51.2	32.2	19.2	7.2	22.4	5.1	NA
	Tonga	14.1 (0.9)	50.3	55.4	26.0	16.2	15.8	9.3	50.6
	Tunisia	13.6 (1.0)	49.7	26.0	9.6	NA	10.6	4.9	30.8
	Vanuatu	13.5 (1.0)	49.5	45.6	12.5	7.6	11.5	15.9	67.9
	Vietnam	14.5 (0.6)	46.6	20.5	3.0	15.5	14.5	4.4	26.1
	Yemen	13.8 (1.0)	56.3	35.1	15.7	NA	27.1	5.9	42.0
Upper middle	Algeria	13.6 (1.1)	45.8	30.1	9.5	NA	NA	NA	51.0
	Antigua & Barbuda	13.9 (0.9)	51.4	30.1	11.8	44.3	16.5	8.4	25.1
	Argentina	13.9 (0.9)	47.7	15.0	19.9	48.1	14.0	5.5	24.4
	Botswana	14.3 (0.8)	46.2	56.0	13.7	20.4	10.7	14.2	52.2
	Costa Rica	14.0 (0.9)	49.6	6.8	10.3	23.3	15.4	5.6	19.1
	Fiji	14.4 (0.6)	49.0	51.3	11.7	13.2	7.7	7.9	30.0
	Grenada	13.7 (1.1)	42.7	28.6	8.1	43.1	18.1	8.3	27.5
	Iraq	13.9 (1.0)	54.7	16.5	12.4	NA	18.0	6.5	28.3
	Lebanon	13.7 (1.0)	46.6	13.3	NA	28.5	12.5	3.6	24.9
	Libya	13.6 (1.0)	49.2	45.6	7.0	NA	20.6	NA	35.4
	Malaysia	14.0 (0.9)	49.5	32.2	10.9	7.5	18.7	3.2	21.0
	Mauritius	13.8 (1.0)	49.2	12.6	16.1	23.5	NA	NA	35.2
	Namibia	14.1 (0.9)	42.9	48.1	11.6	23.0	11.8	13.2	45.9
	Oman	13.9 (0.9)	51.5	26.5	NA	NA	10.6	NA	38.7
	Peru	14.1 (0.8)	49.9	18.7	17.7	26.9	16.4	5.5	47.2
	Seychelles	13.6 (1.1)	49.9	43.0	19.2	57.1	13.2	5.8	52.7
	St. Vincent & the Grenadines	13.5 (1.0)	46.2	29.9	11.3	49.1	14.1	8.2	30.1
	St. Lucia	13.7 (1.1)	44.5	26.2	11.4	52.6	14.1	8.6	25.6
	Suriname	14.0 (1.0)	45.4	23.0	10.0	31.2	12.7	15.8	26.2
	Thailand	13.7 (1.0)	49.6	31.9	13.1	17.6	16.8	5.9	32.7
	Tuvalu	13.3 (1.1)	48.9	22.0	18.6	10.9	37.2	16.2	30.1
	Uruguay	13.8 (1.1)	45.1	6.3	17.1	55.2	6.9	2.8	22.6

Abbreviation: FI Food insecurity, PS/M Parental support/monitoring; NA Not available

All data are percentage apart from age [mean (standard deviation)].

## Results

The final sample consisted of 181,793 adolescents aged 12–15 years with a mean (SD) age of 13.8 (1.0) years and 49.0% were girls. The overall prevalence of ≥3 hours/day of LTSB was 26.4% (95%CI = 25.6%-27.2%), and this prevalence ranged widely between countries with the range being 8.2% (Pakistan) to 54.6% (Antigua & Barbuda) (Table 1).The country-wise mean age and prevalence of each of the other correlates are illustrated in Table 2. The associations between each correlate and ≥3 hours/day of LTSB estimated by meta-analysis based on county-wise estimates are shown in Table 3. In the overall sample, increasing age (OR = 1.14; 95%CI = 1.11–1.17), smoking (OR = 1.85; 95%CI = 1.69–2.03), alcohol consumption (OR = 2.01; 95%CI = 1.85–2.18), and bullying victimization (OR = 1.39; 95%CI = 1.31–1.48) were positively associated with higher LTSB while food insecurity (OR = 0.93; 95%CI = 0.89–0.97) and low parental support/monitoring (OR = 0.91; 95%CI = 0.85–0.98) were negatively and significantly associated with LTSB. These significant associations were observed across country-income levels with some exceptions, i.e., non-significant associations for food insecurity in low-income and upper middle-income countries, and low parental support/monitoring in lower and upper middle-income countries. Sex and having close friends were not significantly associated with LTSB in the overall sample or samples by country-income level. The between-country heterogeneity as estimated by Higgin’s *I*^*2*^ was high for most associations. The country-wise estimates are shown in S1–S8 Figs of the online only supplement. It is worth noting that despite the fact that overall, sex was not significantly associated with LTSB, country-wise analyses showed there were some countries where significant associations were observed. For example, males were significantly more likely to engage in LTSB in countries such as Kenya, Senegal, Myanmar, India, Samoa, Egypt, Algeria, and Tuvalu, while significantly higher odds of LTSB for girls was observed in Mozambique, Tunisia, Belize, Mongolia, Antigua & Barbuda, Costa Rica, Argentina, and Uruguay (S2 Fig).

**Table 3:** Association between each correlate and ≥3 hours/day of leisure-time sedentary behavior estimated by meta-analysis based on country-wise estimates.

		Overall		Low-income		Lower middle-income		Upper middle-income	
Correlate		OR (95%CI)	*I*^*2*^	OR (95%CI)	*I*^*2*^	OR (95%CI)	*I*^*2*^	OR (95%CI)	*I*^*2*^
Age (year)	per one year increase	**1.14 (1.11–1.17)**	59.5	**1.11 (1.03–1.19)**	56.7	**1.12 (1.09–1.16)**	42.0	**1.17 (1.12–1.22)**	71.3
Sex	Male vs. Female	0.99 (0.94–1.04)	65.9	1.06 (0.90–1.24)	67.2	1.00 (0.92–1.08)	64.6	0.95 (0.88–1.02)	63.0
Food insecurity	Yes vs. No	**0.93 (0.89–0.97)**	49.6	0.98 (0.87–1.09)	15.1	**0.91 (0.85–0.98)**	55.1	0.93 (0.87–1.00)	53.3
Smoking	Yes vs. No	**1.85 (1.69–2.03)**	79.1	**2.10 (1.61–2.74)**	71.2	**1.92 (1.71–2.15)**	65.8	**1.69 (1.44–1.99)**	86.6
Alcohol consumption	Yes vs. No	**2.01 (1.85–2.18)**	77.1	**2.23 (1.68–2.97)**	78.6	**2.18 (1.92–2.48)**	71.5	**1.76 (1.60–1.93)**	67.7
Low parental support/monitoring	Yes vs. No	**0.91 (0.85–0.98)**	64.4	**0.69 (0.57–0.82)**	33.8	0.90 (0.82–1.00)	66.6	1.01 (0.92–1.11)	51.6
Close friends	None vs. At least one	0.97 (0.91–1.04)	34.1	0.86 (0.75–1.00)	9.0	1.02 (0.95–1.11)	9.1	0.95 (0.85–1.07)	56.6
Bullying victimization	Yes vs. No	**1.39 (1.31–1.48)**	73.6	**1.55 (1.27–1.88)**	73.1	**1.41 (1.31–1.53)**	55.2	**1.30 (1.18–1.44)**	80.2

Abbreviation: OR Odds ratio; CI Confidence interval

Statistically significant associations (P<0.05) are highlighted in bold font.

Estimates were obtained by combining country-wise estimates adjusted for age, sex, and food insecurity into a meta-analysis with random effects.

## Discussion

This multinational study including 181,793 adolescents aged 12–15 years from 66 LMICs demonstrated that increasing age, smoking, alcohol consumption and bullying victimization were positively associated with LTSB, while a negative association was observed for food insecurity and low parental support/monitoring. These significant associations were almost consistently observed across country-income levels. The only exceptions were food insecurity and parental monitoring/support for which a significant association was not observed across all country-income levels. Sex and having close friends were not significantly associated with LTSB. In summary, our data show that at both the personal and interpersonal level of the socio-ecological model of sedentary behavior [11], significant correlates can be found.

At individual country level, there were some differences in the direction of the associations at the personal level of the socio-ecological model of sedentary behavior [11], with a high level of between-country heterogeneity being observed for most associations. For example, while in some countries boys were more sedentary (e.g., Myanmar, Egypt, Tuvalu), in other countries, girls spend more time sedentary (e.g., Mozambique, Yemen, Antigua & Barbuda). It is known that in some cultures parents are less likely to allow girls to be physically active outdoors, while boys are more likely to engage in outdoor sports activities (e.g. soccer)[28]. Relatedly, while most children and young adolescents are allowed to go out without an adult, in some cultures, particularly girls are only allowed with peers [29]. Consequently, boys, who are allowed to go out without an adult may go out more often after school, and so have more chance to be active and sociable [29]. These differences suggest that culturally defined gender roles are likely to be an important factor when considering lifestyle behaviors in adolescents in LMICs. Therefore, further research needs to explore these cultural factors within specific LMICs to ensure culturally appropriate, tailored intervention strategies can be developed and implemented.

Also at the personal level of the socio-ecological model of sedentary behavior [11], food insecurity was inversely associated with LTSB. Although the exact mechanisms underlying this finding are also here unclear, it is possible that adolescents in food insecure households need to assist their parents after school hours in income generating activities [30]), resulting in a negative relationship.

Finally at the personal level of the socio-ecological model [11], lifestyle factors should be considered when developing interventions focusing on reductions in LTSB, in particular smoking and alcohol consumption, even in young adolescents aged 12 to 15 years in LMICs. Our data confirm that also in adolescents in LMICS, unhealthy lifestyle habits commonly co-occur as clusters. Therefore, multiple health behavior change interventions should be developed in which shared risk factors are targeted together, rather than in isolation. This is particularly important in adolescents as many lifestyle risk behaviors emerge and develop in this period of life and then persist into adulthood [31]. The adoption of a healthy lifestyle in adolescence can therefore have protective effects against the onset of chronic disease [32]. Although challenging to implement in low-income settings, multiple health behavior change interventions might have the potential to achieve this in an efficient and cost-effective manner[33].

At the interpersonal level of the socio-ecological model of sedentary behavior [11], an inverse association between low parental support/monitoring and high levels of sedentary behavior (or in other words, high levels of parental support/monitoring are associated with higher likelihood of higher levels of sedentary behavior) was observed. However, parental support/monitoring was only associated with higher likelihood of being more sedentary in low-income countries. It might be that parents in low-income countries who are involved in what their children are doing during their after school leisure time are more worried about the dangers of playing outside in an often unsafe and polluted environment and prefer that their children remain inside.

Interpersonal factors may be important and modifiable variables, therefore, health campaigns should focus on these when formulating behavior change strategies or campaigns [34]. Another interpersonal factor associated with more sedentary behavior in adolescents aged 12–15 years in LMICs is bullying victimization. This finding stresses the importance of bullying prevention efforts in conjunction with health promotion programs targeting school going adolescents. A factor that might mediate the relation between bullying victimization and sedentary behavior is low mood [35]. Previous research has shown that bullying victims experience severe emotional distress and depression associated with the psychological and physical violence they are subjected to [36]. Emotional distress and depression are in turn associated with more time spent sedentary [37]. Bullying is also shown to negatively influence self-efficacy [38]. Lower self-efficacy has been associated with specific sedentary behaviors such as time spent watching DVDs and using the computer for non-school purposes in adolescents [39]. An additional component is that bullying is associated with social isolation and subsequently increased LTSB [18]. Thus, inclusive approaches to reduce social isolation and LTSB could be a promising approach.

Finally, at the interpersonal level of the socio-ecological model of sedentary behavior [11], having close friends was unrelated to levels of sedentary behavior in adolescents aged 12–15 years from LMICs. Rather than the number of close friends, the strength of the relationship with best friends might be of relevance when considering how friends influence each other’s sedentary behavior [40]. To the best of our knowledge, processes of peer influence on sedentary behavior including behavioral modeling (i.e., observing a friend being sedentary leading to increased likelihood of becoming sedentary as well) or group norms (i.e., the underlying attitudes and behaviors shared among a group of friends) have not been explored yet in LMICs. To this end, social network analysis [41] provides a way of investigating the relationships among friends themselves. Social network analysis does not rely on an individual recalling or reporting the behavior, but is a quantitative method for assessing the structure and patterns of the relationships among adolescents [41]. Given the increasing use of digital technologies in LMIC settings, and close relationships between real-world and online social networks [42], future research should consider applying social network analysis to examine the interactions between screen time, social networks, sedentary behaviors and other health risks in LMIC settings.

The current findings should be interpreted in light of some limitations. First, due to the cross-sectional design, the cause-effect relationships remain unclear. Longitudinal studies are required to better disentangle the relationships observed. Second, we used the GSHS data, which surveys only adolescents in schools and may not reflect LTSB among all adolescents. Third, sedentary behavior was also only captured with a self-report measure. The accuracy of self-reported physical activity has been questioned in adolescents[43]. Besides this, time spent sedentary excluded time at school and when doing homework. It is therefore an underestimate of the real time spent sedentary during the entire day. Given the recent mass-scale adoption and regular usage of smartphones among young people, also in LMICs [42], activity trackers on smartphones may present novel and feasible methods for collecting more objective measures of sedentary behavior on a population-scale. Future studies may benefit as well from distinguishing between passive and mentally-active sedentary behaviors, which are shown to have differential effects on mental health in adults [44]. Finally, possible reasons for the country-wise differences could be urbanization rate such that participants from rural areas might be less sedentary than adolescents from urban areas [45]. We however did not have data about whether adolescents were living in urban or rural areas.

Nonetheless, the strengths of the study include the largest sample size to date on this topic and the multi-national scope across LMICs. Most of the research in the domain of sedentary behavior has been conducted in high-income countries, and little is known about regions where there are multiple economic, cultural or social factors or differences in the health systems. The present study was furthermore performed with nationally representative samples of adolescents attending school.

In conclusion, our data indicate that in adolescents aged 12 to 15 years living in LMICs, sedentary behavior is a complex and multi-dimensional behavior determined by modifiable sociocultural and lifestyle factors. Future longitudinal data are required to confirm/refute the findings to inform interventions which aim to decrease sedentary levels in adolescents living in LMICs.

## Supporting information

S1 Fig Country-wise association between age (exposure) and ≥3 hours/day of leisure-time sedentary behavior (outcome) estimated by multivariable logistic regression. (DOCX)

S2 Fig Country-wise association between sex (male vs. female) (exposure) and ≥3 hours/day of leisure-time sedentary behavior (outcome) estimated by multivariable logistic regression. (DOCX)

S3 Fig Country-wise association between food insecurity (exposure) and ≥3 hours/day of leisure-time sedentary behavior (outcome) estimated by multivariable logistic regression. (DOCX)

S4 Fig Country-wise association between smoking (exposure) and ≥3 hours/day of leisure-time sedentary behavior (outcome) estimated by multivariable logistic regression. (DOCX)

S5 Fig Country-wise association between alcohol consumption (exposure) and ≥3 hours/day of leisure-time sedentary behavior (outcome) estimated by multivariable logistic regression. (DOCX)

S6 Fig Country-wise association between low parental support/monitoring (exposure) and ≥3 hours/day of leisure-time sedentary behavior (outcome) estimated by multivariable logistic regression. (DOCX)

S7 Fig Country-wise association between close friends (none vs. at least one) (exposure) and ≥3 hours/day of leisure-time sedentary behavior (outcome) estimated by multivariable logistic regression. (DOCX)

S8 Fig Country-wise association between bullying victimization (exposure) and ≥3 hours/day of leisure-time sedentary behavior (outcome) estimated by multivariable logistic regression. (DOCX)
