# Supplementary material for: Correlates of leisure-time sedentary behavior among 181,793 adolescents aged 12-15 years from 66 low- and middle-income countries
Source: PLoS One. 2019 Nov 14;14(11):e0224339. doi: 10.1371/journal.pone.0224339 (PMC6855478; doi:10.1371/journal.pone.0224339)
Supplement: S6 Fig — (DOCX) [file pone.0224339.s006.docx]

**S6 Fig** Country-wise association between low parental support/monitoring (exposure) and ≥3 hours/day of leisure-time sedentary behavior (outcome) estimated by multivariable logistic regression

**S6 Fig** Country-wise association between low parental support/monitoring (exposure) and ≥3 hours/day of leisure-time sedentary behavior (outcome) estimated by multivariable logistic regression

Abbreviation: OR Odds ratio; CI Confidence interval.

Models are adjusted for age, sex, and food insecurity.

Overall estimate was obtained by meta-analysis with random effects.
